# Four kinds of traditional Chinese exercise therapy in the treatment of type 2 diabetes: a systematic review and network meta-analysis

**DOI:** 10.1186/s13643-023-02384-1

**Published:** 2023-12-13

**Authors:** Yuqi Jia, Hailiang Huang, Ying Yu, Hongling Jia, Yongchen Zhang

**Affiliations:** 1 College of Acupuncture and Massage, Shandong University of Traditional Chinese Medicine, Jinan, China; 2 College of Rehabilitation Medicine, Shandong University of Traditional Chinese Medicine, Jinan, China; 3 Institute of Chinese Medicine Innovation, Shandong University of Traditional Chinese Medicine, Jinan, China; 4 Second Affiliated Hospital, Shandong University of Traditional Chinese Medicine, Jinan, China

## Abstract

**Objective:**

To perform an evidence-based evaluation of the clinical efficacy of Taijiquan, Baduanjin, Yijinjing and Wuqinxi in interventions for type 2 diabetes.

**Design:**

A systematic review and network meta-analysis.

**Methods:**

The comprehensive search included Chinese and other language databases such as the MEDLINE (PubMed), Web of Science, Excerpta Medica Database (Embase), The Cochrane Library, China National Knowledge Infrastructure, Wanfang Data Knowledge Service Platform, China Scientific Journal Database, VIP and China Biomedical Literature Database (CBM). Clinical randomized controlled trials of four traditional Chinese exercise therapies in the treatment of type 2 diabetes, including Taijiquan, Baduanjin, Yijinjing and Wuqinxi, were retrieved. The search time was conducted from the establishment of the database to 30 October 2022. Two researchers screened the documents that met the inclusion criteria, extracted data according to the preset table and evaluated the methodological quality of the included studies according to the quality evaluation tools recommended by the Cochrane System Reviewer Manual V.5.1. The R language, Stata and ADDIS statistical software programs were used to conduct statistics and analysis of intervention measures.

**Results:**

A total of 33 randomized controlled trials with 2609 patients were identified. All patients were from China. The results of the network meta-analysis showed that Taijiquan ranked the best for improving HbA1c, 2-h postprandial blood glucose (2hPG), low-density lipoprotein cholesterol (LDL-C) and insulin sensitivity index indicator levels; Yijinjing reduced fasting plasma glucose (FPG) and total cholesterol (TC) indicator levels for the best probability ranking; Baduanjin improved the triglyceride (TG) and high-density lipoprotein cholesterol (HDL-C) probability ranking the most. When the training period was less than 12 weeks, Baduanjin had better effects in improving 2hPG, TC, TG, HDL-C and LDL-C indicator levels. Taijiquan had better effects in reducing FPG levels. When the training period was 12 weeks, the effect of Yijinjing in improving FPG, HbAlc, TC and HDL-C levels was better than that in other traditional Chinese exercise, and Taijiquan had better effects in improving 2hPG, TG and LDL-C indicator levels. When the training period was longer than 12 weeks, Taijiquan had better effects in improving FPG, HbAlc, 2hPG and LDL-C indicator levels, and Baduanjin had better effects in improving TC, TG and HDL-C indicator levels.

**Conclusion:**

The four traditional Chinese exercise therapies can improve blood glucose levels, blood lipid levels and insulin-related indicators of type 2 diabetes to varying degrees. Studies have shown that Taijiquan has a better targeted treatment effect on type 2 diabetes.

**Systematic review registration:**

CRD42020214786.

**Protocol published:**

We published the protocol article “Network meta-analysis of four kinds of traditional Chinese exercise therapy in the treatment of type 2 diabetes: Protocol for a systematic review” in the *BMJ Open* magazine 2021, Issue 11, Volume 7.

## Introduction

Type 2 diabetes (type 2 diabetes mellitus, T2DM), also known as non-insulin-dependent diabetes, is the most common form of clinical diabetes and mainly manifests as persistent hyperglycaemia. Its aetiology and pathogenesis are not yet clear, but the disease is mainly related to insulin secretion defects related to inflammation and metabolic stress, including the involvement of genetic factors [1]. The significant pathophysiological features of type 2 diabetes are the decrease in insulin’s ability to regulate glucose metabolism (insulin resistance) and the decrease (or relative decrease) in insulin secretion caused by defects in pancreatic islet B cell function [2]. As the natural course of the disease develops, the dependence on exogenous blood sugar control increases, which can affect multiple tissues and organs and can cause a variety of secondary complications [3]. The disease can affect the large blood vessels (cerebrovascular disease, cardiovascular disease), microvasculature (podiatry disease, eye disease, kidney disease) and nerves (nephropathy, eye disease) [4], reducing the quality of life of patients and endangering patients’ lives.

With changes in living standards and environments, the incidence of T2DM is increasing [5]. The 10th edition of the Global Diabetes Map shows that 783.2 million people worldwide are expected to have diabetes by 2045, with type 2 diabetes accounting for more than 90% of the total diabetic population [6]. At present, China has become the country with the largest number of diabetes patients in the world, with the number of people affected reaching 140.9 million, which has a huge impact on human life [7]. The main methods of blood sugar control in T2DM patients involve oral hypoglycaemic drugs and exogenous insulin supplements. Traditional treatment is highly dependent on drugs, and oral drugs and exogenous insulin can only temporarily maintain blood sugar or temporarily improve insulin sensitivity and are not curative [4].

According to the latest version of the domestic guidelines for the Prevention and Treatment of Type 2 Diabetes (2017 edition), lifestyle intervention is the basic treatment measure for T2DM and is required for the treatment of diabetes; exercise plays an important role in the comprehensive management of T2DM patients [3]. According to the guidelines of the American College of Sports Medicine and the American Diabetes Association, it is recommended that diabetes patients perform aerobic exercise. Patients with type 2 diabetes should perform at least 150 min of moderate- or high-intensity exercise per week, and they should perform it at least 3 days per week [8]. Long-term regular aerobic exercise can improve the body weight and blood sugar and blood lipid levels of type 2 diabetes patients and has an important role in the rehabilitation of type 2 diabetes patients and the prevention of complications [9].

Taijiquan is a kind of traditional Chinese boxing which combines both internal and external cultivation and the combination of hardness and softness [10]. At present, the simplified 24-form Taijiquan, which has been rearranged, is widely accepted by the masses [11]. Baduanjin is a set of independent and complete fitness exercises, and this exercise a total of eight movements, practice without equipment, not limited by the site, with soft and slow, static and Qi in the characteristics [12]. Wuqinxi is an important exercise in traditional Chinese guiding health. It has ten movements, imitating the movements of five animals, such as tiger, deer, bear, ape and bird, and combining exhalation and inhalation with internal and external integration, so that the joints, muscles and muscles of the whole body can be stretched. It is a medium and small-intensity aerobic exercise [13]. Yijinjing has 12 movements, and during the exercise process, the bones and joints of the human body show multi-directional and wide-angle activities as much as possible, promote the movement of human qi and blood, enhance the strength of the limbs and improve the physiological functions of various tissues and organs of the human body [14].

The four traditional Chinese exercise therapies described above conform to the characteristics of a low-intensity and long-term aerobic exercise proposed by modern research. It is guided by the holistic concept of Chinese medicine as the main theoretical guide. By mobilizing the human body’s own potential, it can achieve the purpose of healing and strengthening the body and preventing and curing diseases, and it is a nondrug therapy for T2DM [15, 16]. In recent years, a number of randomized controlled studies have shown that the four traditional exercise therapies play an irreplaceable role in the prevention of T2DM at all levels. Studies have found that Taijiquan can reduce damage to pancreatic islet cells by downregulating the expression of inflammatory cytokines, improving the body’s sensitivity to insulin, improving insulin resistance, delaying the occurrence of diabetes complications and improving the quality of life of patients with T2DM [17]. The application of Baduanjin in populations at high risk of diabetes can delay the onset of glucose metabolism disorders, exert good stabilizing effects on blood sugar and glycosylated haemoglobin levels and reduce regulatory fluctuations [18]. Wuqinxi exercise therapy can significantly improve the blood rheology of patients with T2DM and improve blood circulation function [19]. Yijinjing can effectively regulate liver and spleen function in patients with T2DM and can help improve blood sugar levels [20].

At present, studies in this area mostly use randomized controlled trials to verify the clinical efficacy of a single traditional exercise therapy on T2DM, and there is no evidence-based evaluation that compares the clinical efficacy of the four traditional exercise therapies for T2DM concurrently. Therefore, this study selected four traditional exercise therapies commonly used in clinical practice as the research objects and used the method of network meta-analysis to integrate relevant clinical evidence. After summarizing the different interventions in the same body of evidence, a quantitative comprehensive statistical analysis was performed to compare the clinical efficacy of four different traditional Chinese exercise therapies in the treatment of T2DM. Our purpose is to provide a reference for the clinical treatment of T2DM to provide more effective exercise intervention therapies.

## Methods and analysis

### Patient and public involvement

No patients were involved.

### Eligibility criteria

The design of the inclusion and exclusion criteria of this study was based on the five main principles of the Participant-Intervention-Comparator-Outcomes-Study (PICOS) design search principle.

### Inclusion criteria

#### Type of participants

The included patients all had type 2 diabetes, regardless of age, sex and race. The diagnostic criteria used for diabetes complied with the “China Type 2 Diabetes Guidelines (2010 Edition)” [21], the “China Type 2 Diabetes Prevention Guidelines (2013 Edition)” [22], the “China Type 2 Diabetes Prevention Guidelines (2017 Edition)” [3], the “Diagnostic Guidelines for Diabetes Diagnosis and Classification Standards” (revised by the WHO in 1997) [23] and the “Diagnostic Standards for Diabetes” (created by the WHO in 1999).

### *Types of *interventions* and comparators*

The control group received conventional basic treatment (e.g. hyperglycaemic treatment, health education, voluntary exercise) without traditional exercise intervention. The treatment group was treated with one of the four traditional exercise therapies of Taijiquan, Baduanjin, Yijinjing and Wuqinxi when the diagnostic criteria, curative effect evaluation criteria and basic treatment were the same.

### Types of outcomes

The main outcome indicators were as follows: blood glucose levels, including fasting blood glucose (FBG, fasting plasma glucose (FPG)), 2-h postprandial blood glucose (2hPG, PPG) and glycosylated haemoglobin (GHb, HbA1c).

The secondary outcome indicators were as follows: (a) blood lipid levels, including total cholesterol (TC) and triglycerides (TG), high-density lipoprotein cholesterol (HDL-C) and low-density lipoprotein cholesterol (LDL-C), and (b) insulin levels, including insulin sensitivity index (ISI).

### Types of studies

The included literature type was randomized controlled trials (RCTs), and there were no restrictions regarding the type of language, whether blinding was used, or the requirements for allocation concealment. As long as the included study was approved by the local institution, we included the study in the scope of this study and registered it in the international database.

### *Exclusioncriteria*

Self-control studies, case reports, literature reviews, duplicate publications, summaries of experiences, animal experiment research, studies with incomplete data, studies with patients who had other diseases, studies with no clear diagnosis or efficacy evaluation standard and studies that combined other therapies that were different from the control group were excluded.

### Search methods for the identification of studies

#### Information sources

A computer was used to conduct a comprehensive search for four traditional randomized controlled trials (RCTs) for the treatment of type 2 diabetes. The search time was from the establishment of the database to October 30, 2022. Computer databases that were searched included PubMed, Web of Science, Embase, the Cochrane Library, China Knowledge Network Infrastructure (CNKI), Wanfang Data Knowledge Service Platform, VIP.com (VIP) and China Biomedical Literature Database (CBM). Search terms included Taijiquan, Baduanjin, Yijinjing, Wuqinxi, traditional exercises, Health Qigong, type 2 diabetes and random.

In addition, relevant references were tracked in the literature, and the corresponding authors were contacted when a complete report could not be obtained or when documents included incomplete relevant data. The best effort was made to ensure the comprehensiveness of the preliminary search work so as not to lose valuable research data. According to the search modes of different databases, keywords could be combined with free words for a comprehensive search.

### *Study selection *and* data extraction*

According to the abovementioned electronic database search strategy, two researchers searched the Chinese and English electronic databases, used EndNote X7 software to search for repeated studies, integrated the literature search results of the different databases, established an information database and downloaded the full texts. Then, two researchers conducted preliminary screening independently, extracted data according to a predetermined table, conducted cross-checking and review, recorded the reasons for each excluded study and invited third-party experts to discuss and research different opinions to make the final decision.

The data extraction content included the basic information of the included literature (including the first author, publication journal and year and research topic), the relevant information of the experimental group and the control group in the literature (including the number of cases, disease course, age, intervention measures, treatment course and outcome indicators) and the design type and the quality evaluation information of the included literature (e.g. random method, blind method, allocation concealment, completeness of outcome data, selective reporting results, other sources of bias).

### Study quality evaluation

The methodological quality of included studies was evaluated using Cochrane’s revised Cochrane risk of bias tool for randomized trials (RoB 2.0), including randomization process, deviations from intended interventions, missing outcome data, measurement of the outcome and selection of five areas of the reported result. Each module to be evaluated contains multiple signal questions, and the alternative answers to the signal questions include the following: Y (yes), PY (probably yes), PN (probably no), N (no) and NI (no information). Based on the above description, the two researchers conducted an individual induction study to determine the quality evaluation results of the included literature. If the results are different, a third-party expert is invited to help discuss and explain the quality assessment. Cochrane’s standard manual was used for the literature quality evaluation and bias risk assessment [24, 25].

### Data synthesis and statistical methods

#### Pairwise and network meta-analysis

First, the authors used RevMan software to analyse the direct comparison results of the literature. Second, for the indirect comparison results, the authors used R and ADDIS software for data merging, statistical analysis and network meta-analysis (NMA) while drawing a network relationship diagram and anecdotal sequence diagram of the various intervention measures [26]. The netmeta program was launched using the R programming language, and the Bayesian Markov chain Monte Carlo (MCMC) algorithm was invoked through the relevant commands to perform the network meta-analysis and the best probability ordering for comparing the differences between the four different exercise therapies, and to realize the mapping between the network data analysis and the results of the random-effects model data. ADDIS statistical software uses relevant instructions to call the data results of the random-effects model based on the Bayesian MCMC algorithm for prior evaluation and processing.

*P* < 0.05 and 95% confidence intervals (95% CIs) were used as the standards of significant difference, and the count data used the OR value as the efficacy analysis statistic; the measurement data used the weighted mean difference or the standardized mean difference (mean difference, MD) and indicated that each effect size was expressed with a 95% CI.

### Assessment of heterogeneity

The heterogeneity was graded using *I*^2^ according to the recommendations of the Cochrane Handbook. The *I*^2^ index was used for the statistical heterogeneity assessment, and *x*^2^ was used for the subgroup analysis based on heterogeneity factors. The clinical and methodological heterogeneity of the included studies was evaluated, and the levels of fit of the fixed-effects model and the random-effects model were compared. In the absence of significant clinical heterogeneity (*P* ≥ 0.1, *I*^2^ ≤ 50%), a fixed-effects model was used for meta-analysis. If there was significant clinical heterogeneity between the results of each study (*P* < 0.1, *I*^2^ > 50%), the source of the heterogeneity was first analysed, the influence of clinical or methodological heterogeneity was excluded and the random-effects model was used for the meta-analysis. When the data provided by the clinical trial could not be meta-analysed, they were subjected to a descriptive analysis [27].

### Subgroup and sensitivity analyses

If there was heterogeneity and the data were sufficient, we tried to use subgroup analysis to determine the reasons for the heterogeneity and compare the effects of each group. Data could be compared between patients of different sexes, ages, courses of disease and treatment times. If the result of the meta-analysis was positive and more than three studies were included, R software was used to perform a sensitivity analysis of the statistical results. For each excluded study, the meta-analysis was performed again, and the results were compared with the results before exclusion. If there was no substantial change in the comparative analysis, the results were stable. Otherwise, the data results were unstable.

### Assessment of inconsistency

The node-split model was used for inconsistency testing. If there was no significant difference between the studies within the subgroup (*P* > 0.05), the heterogeneity of the included studies was small, so the consistency model was used for analysis. Otherwise, the inconsistency model was used for analysis. ADDIS software mainly evaluates the final iterative effect of the interchain and intrachain variances through the convergence of the model, that is, the subsequent evaluation through the potential scale reduced factor (PSRF) parameters. The recommended use in this software is to limit the PSRF value, which is more reasonable between 1 and 1.05. If the PSRF value is not very close to 1, expansion of the model can continue. Research through software analysis calculations and data analysis found that the PSRF value was close to or equal to 1, indicating that good convergence performance was achieved, and the results obtained from the consistency model analysis were more reliable [28].

### Publication bias

According to the recommendations of the Cochrane Handbook, if more than ten studies were included, RevMan software was used to analyse potential publication bias. If the graph showed inverted funnel-like symmetry, it indicated that the possibility of publication bias was relatively small. If the funnel chart was asymmetric or incomplete, it indicated that there was a greater possibility of publication bias.

## Results

### Literature screening process and results

**Fig. 1:**
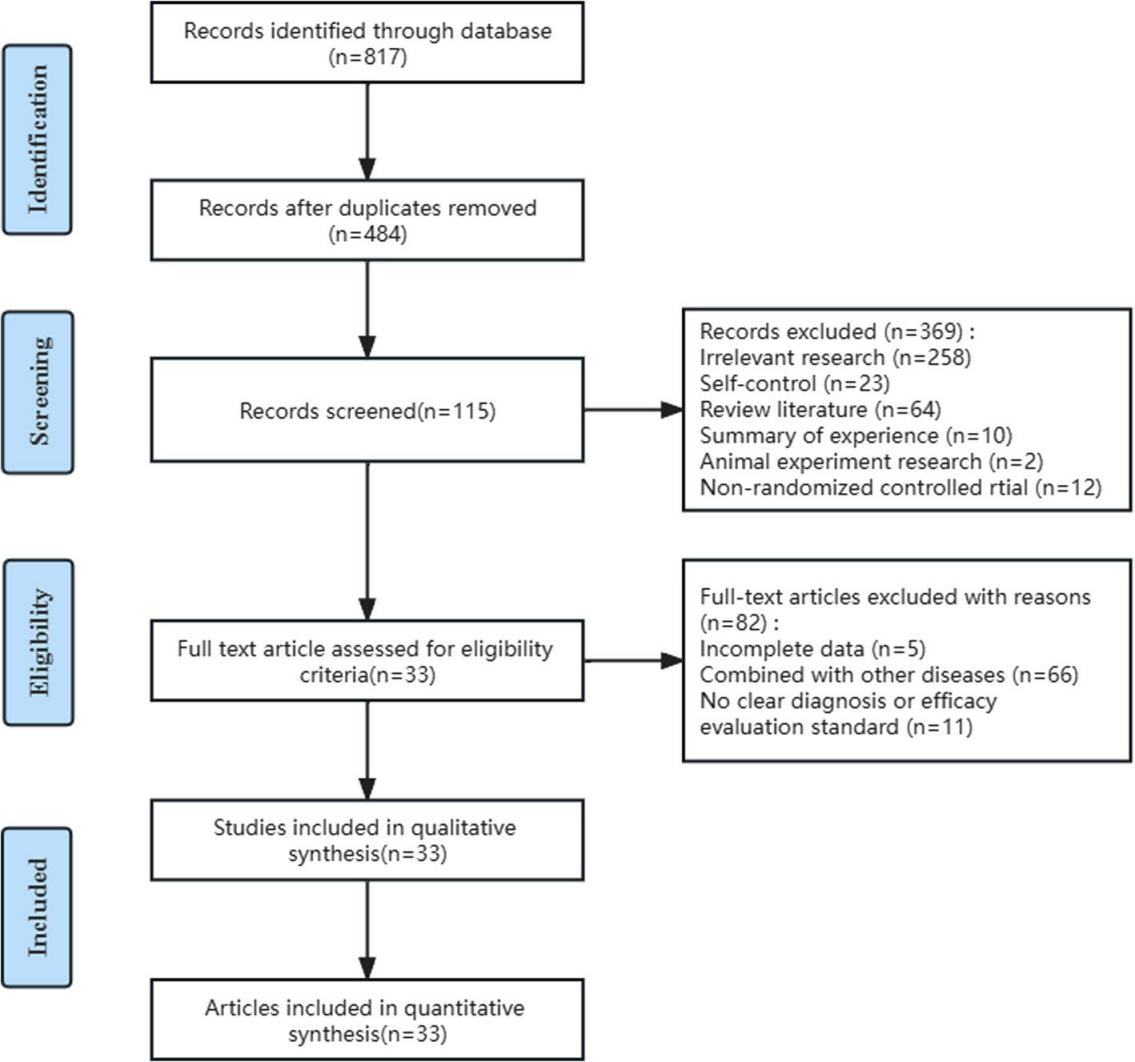
Flow chart of the preferred reporting project for the systematic review and network meta-analysis

A total of 817 studies were obtained from the initial search. After strict and careful screening, 33 studies were finally included. The report flowchart is shown in Fig. 1.

### Study characteristics

After screening, a total of 33 studies were included. Most of the clinical trial reports in this study were from Mainland China, and the patients eventually included in RCTs were all from China. The treatment group included 13 Taijiquan exercises, 18 Baduanjin exercises, 1 Wuqinxi exercises and 2 Yijinjing exercises each. A total of 2609 patients were enrolled, of which 1295 were included in the control group, 1314 were included in the treatment group and 30 dropped out/were lost to follow-up. The basic characteristics of the included studies are shown in Table 1.

**Table 1:** Characteristics of the selected studies included in the systematic review and network meta-analysis

Author (year)	TG			CG			Treatment cycle	Outcome indicators^a^
	Sample size	Age (years)	Treatment measures	Sample size	Age (years)	Treatment measures		
Kan (2004)	26	52 ± 6.7	T	22	52 ± 6.7	N	12	①②③④⑤
Zhang (2008)	10	57.4 ± 6.2	T	9	57.4 ± 6.2	N	14	①②③④⑤
Wu (2010)	20	51.3 ± 7.9	T	20	52.4 ± 5.5	N	24	①⑥⑦
Chen (2010)	50	56.1 ± 6.2	T	44	57.4 ± 5.8	N	12	①②③⑤⑥
Ji (2012)	32	60.31 ± 7.23	B	30	60.26 ± 7.15	N	8	①⑥⑧
Sununta (2013)	32	35 ± 5.63	T	32	36.16 ± 4.48	N	12	①⑥
Li (2013)	30	57.3 ± 10.3	T	30	57.3 ± 10.3	N	8	①⑧
Li (2013)	54	54.21 ± 9.47	T	54	50.42 ± 9.68	B	12	①③⑤⑥
Meng (2014)	100	68.4 ± 3.2	T	100	68.4 ± 3.2	N	12	①②③④⑤⑥⑧
Li (2015)	50	62.91 ± 2.48	T	50	63.27 ± 2.86	N	24	①②③④⑤⑥
Shen (2019)	54	67.8 ± 5.1	T	54	66.2 ± 4.6	N	12	①⑥
Zhou (2020)	46	53.1	T	47	53.1	N	8	①⑥⑧
Li (2020)	40	45–75	W	40	45–75	N	4	②③④⑤⑥
Wang (2007)	40	57.8 ± 7.5	B	39	56.5 ± 6.9	N	24	①②③⑤⑥
Pan (2008)	24	47 ± 7	B	24	45 ± 9	N	24	①③⑤⑥
Lin (2009)	24	59.38 ± 5.29	B	23	55.30 ± 8.67	N	16	①⑥
Li (2009)	40	57.8 ± 7.5	B	39	56.5 ± 6.9	N	24	①②③④⑤⑥⑧
Huang (2011)	30	57.8 ± 7.5	B	30	56.5 ± 6.9	N	24	①②③④⑤⑥
Zhou (2011)	61	67.4 ± 9.23	B	61	68.13 ± 10.64	N	12	①②③④⑤⑥⑧
Guan (2012)	39	59.20 ± 8.80	B	40	58.70 ± 8.30	N	16	①②⑥⑧
Duan (2012)	100	47 ± 7	B	100	45 ± 9	N	8	①③⑤⑥
Wang (2015)	30	61.7 ± 6.9	B	30	61.3 ± 8.4	N	6	①②③④⑤⑥⑧
Wu (2015)	20	63.9 ± 7.6	B	20	65.3 ± 6.0	N	12	①⑥
Li (2016)	30	60 ± 3	B	30	59 ± 4	N	12	①⑥⑧
Li (2017)	43	44.32 ± 5.07	B	43	42.91 ± 6.10	N	12	①②③④⑤⑥⑧
Wu (2019)	30	57.8 ± 7.5	B	30	56.5 ± 6.9	N	12	①②③④⑤⑥⑦
He (2019)	24	51.83 ± 14.32	B	24	51.59 ± 11.46	N	48	①②③④⑤⑥
Li (2020)	60	57.42 ± 5.55	B	55	58.89 ± 5.22	N	24	①⑥⑧
Meng (2017)	12	40 ~ 60	Y	12	40 ~ 60	N	12	①②④⑤⑥
Lu (2022)	60	66.5 ± 3.68	Y	60	67.17 ± 2.86	N	12	①②③④⑤⑥
Hu (2022)	30	61.45 ± 4.97	T	30	59.55 ± 6.19	N	12	①②③④⑤⑥⑧
Zhou (2021)	33	62.12 ± 7.59	T	33	60.91 ± 6.99	N	24	①⑥⑧
Qi (2021)	40	41–69	B	40	40–70	N	24	①②③⑤⑥

*TG* treatment group, *CG* control, *T* Taijiquan, *B* Baduanjin, *W* Wuqinxi, *Y* Yijinjing, *N* no traditional exercise intervention

^a^① FBG; ② TG; ③ TC; ④ LDL-C; ⑤ HDL-C; ⑥ HbAlc; ⑦ ISI; ⑧ 2hPG

### Quality assessment and bias risk assessment

**Fig. 2:**
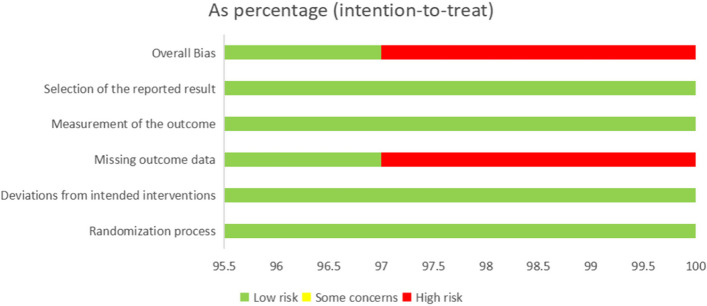
Risk of bias graph in the included studies

**Fig. 3:**
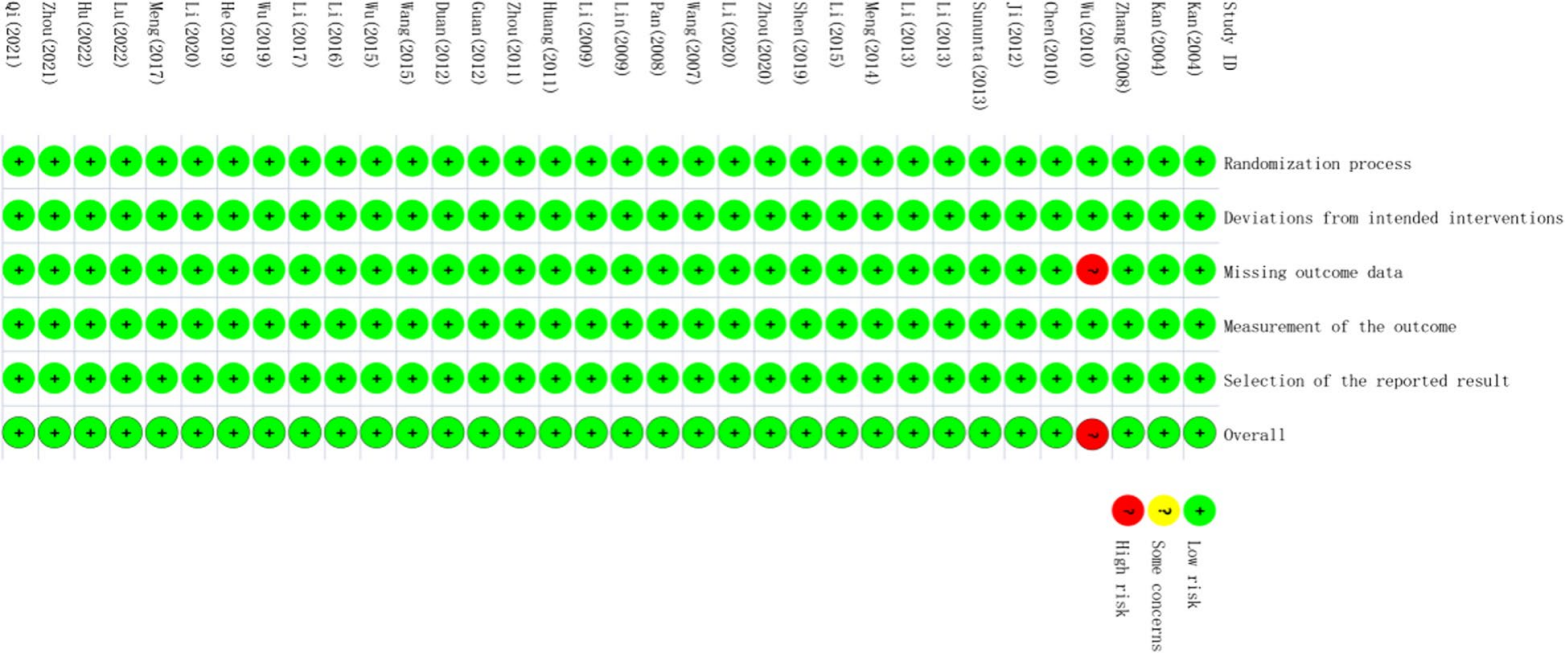
Risk of bias summary in the included studies

In the final overall risk assessment results, 97% were low risk and 3% were assessed as high risk. Among the included studies, 33 were double-arm RCTs. Among them, 13 items were grouped by the random number table method, and 23 items had only random words. Four of the five evaluation modules were rated as low risk. In the missing outcome data evaluation, six studies reported the number of people who dropped out or lost to follow-up, five of which were assessed as low risk based on the RoB 2.0 evaluation results, and only one study was assessed as high risk because it could not determine whether the absence of outcome variables in the lost and shed population was related to the outcome itself. The risk of research bias is expressed as a percentage of all the included studies, as shown in Figs. 2 and 3.

### Consistency and mesh relationship

**Fig. 4:**
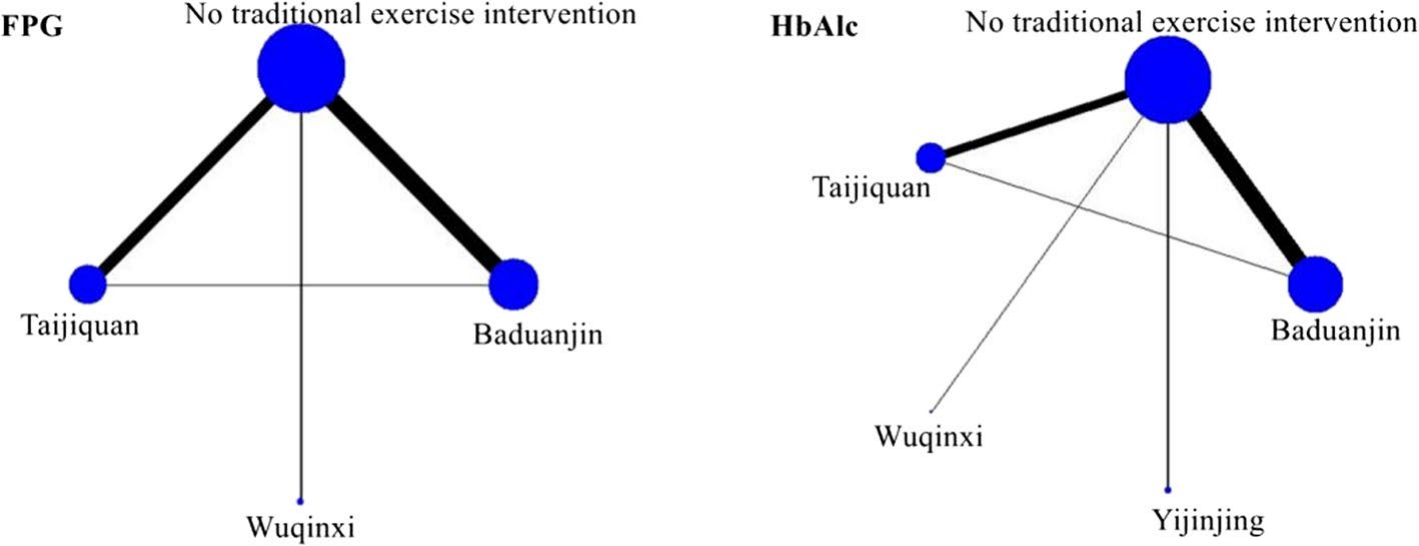
Network diagram of the four kinds of traditional Chinese exercise therapy in the treatment of type 2 diabetes (FPG and HbAlc levels). All of the traditional Chinese exercise therapies used a *P* value < 0.05

Figure 4 shows the network relationship diagram of the four traditional Chinese exercise therapies for type 2 diabetes, represented by the FPG and HbAlc levels. There was a direct comparison between the four traditional exercise therapies and no traditional exercise intervention. There is a direct comparison between Taijiquan and Baduanjin. The size of the circle represents the sample size, and the thickness of the line represents the frequency of the two interventions. Baduanjin was the most researched traditional exercise therapy. Through the analysis, the PSRF value of each outcome index was close to 1 or equal to 1, indicating that good convergence performance was achieved, so the consistency model was used for analysis.

### Results of the network element analysis and subgroup analysis

#### Primary outcome

NMA evaluates a trial comparing traditional Chinese medicine exercise therapy (Taijiquan, Baduanjin, Yijinjing and Wuqinxi) with no traditional exercise intervention therapy. It can indirectly compare the effects of Taijiquan, Baduanjin, Yijinjing and Wuqinxi.

**Fig. 5:**
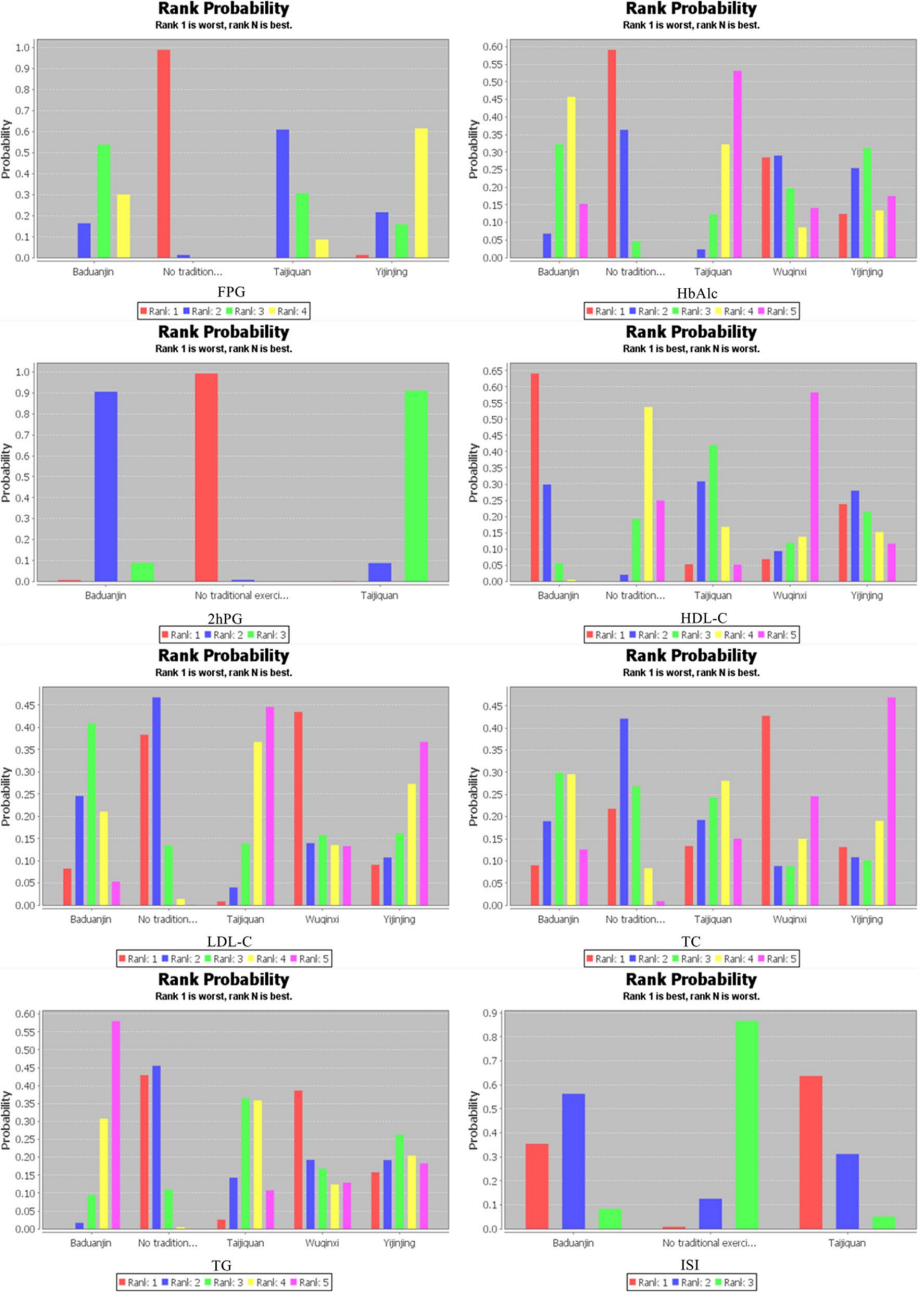
Probability ranking diagram of each outcome indicator

Thirty-two studies reported FPG changes involving three traditional exercise therapies, resulting in a total of six pairwise comparisons. Compared with no traditional exercise intervention group, the MD in the change of FPG levels was 0.49 point lower than that of the Taijiquan group (MD = 0.49; 95% CI 0.33 to 0.69; 1053 participants; *P* < 0.05), 0.41 point lower than that of the Baduanjin group (MD = 0.41; 95% CI 0.31 to 0.55; 1325 participants; *P* < 0.05) and 0.34 point lower than that of the Yijinjing group (MD = 0.34; 95% CI 0.12 to 0.89; 144 participants; *P* < 0.05). Even if the difference between other pairwise comparisons was not statistically significant (*P* > 0.05; Table 2), based on the above meaningful statistical results, we can infer that Yijinjing has the best effect in reducing the FPG index, and the probability ranking is shown in Fig. 5.

**Table 2:** Mesh meta-analysis results

Measure 1	Measure 2	FPG	HbAlc	2hPG	TC	TG	LDL-C	HDL-C	ISI
Taijiquan	Baduanjin	1.18 (0.75, 1.84)	0.83 (0.49, 1.43)	0.43 (0.12, 1.57)	1.34 (0.93, 1.92)	1.6 (0.77, 3.27)	0.88 (0.57, 1.37)	0.91 (0.78, 1.05)	1.17 (0.37, 3.68)
	Wuqinxi	–	0.52 (0.13, 2.11)	–	0.77 (0.34, 1.76)	0.83 (0.18, 3.6)	0.67 (0.29, 1.51)	1.13 (0.81, 1.56)	–
	Yijinjing	1.42 (0.51, 4.14)	0.64 (0.2, 1.99)	–	1.22 (0.5, 2.95)	1.08 (0.33, 3.54)	0.92 (0.47, 1.85)	0.98 (0.76, 1.28)	–
	No traditional exercise intervention	0.49 (0.33, 0.69)*	0.37 (0.24, 0.58)*	0.15 (0.06, 0.42)*	0.76 (0.55, 1.03)	0.73 (0.41, 1.3)	0.65 (0.46, 0.91)*	1.07 (0.94, 1.21)	1.86 (0.81, 4.27)
Baduanjin	Wuqinxi	–	1.59 (0.41, 6.32)	–	1.74 (0.78, 3.88)	1.93 (0.46, 8.21)	1.32 (0.6, 2.92)	0.81 (0.59, 1.1)	–
	Yijinjing	1.21 (0.44, 3.43)	0.77 (0.25, 2.31)	–	0.91 (0.39, 2.16)	0.67 (0.22, 2.09)	1.06 (0.54, 2.04)	1.08 (0.85, 1.38)	–
	No traditional exercise intervention	0.41 (0.31, 0.55)*	0.45 (0.32, 0.62)*	0.36 (0.16, 0.76)*	0.57 (0.45, 0.72)*	0.46 (0.3, 0.72)*	0.74 (0.56, 0.97)*	1.18 (1.08, 1.29)*	1.6 (0.72, 3.57)
Wuqinxi	Yijinjing	–	1.23 (0.22, 6.75)	–	1.58 (0.51, 4.9)	1.3 (0.23, 7.29)	1.39 (0.54, 3.61)	0.87 (0.6, 1.28)	–
	No traditional exercise intervention	–	0.71 (0.19, 2.7)	–	0.98 (0.45, 2.12)	0.89 (0.23, 3.52)	0.97 (0.46, 2.05)	0.95 (0.7, 1.28)	–
Yijinjing	No traditional exercise intervention	0.34 (0.12, 0.89)*	0.58 (0.2, 1.69)	–	0.62 (0.27, 1.42)	0.68 (0.24, 1.92)	0.7 (0.38, 1.26)	1.09 (0.86, 1.37)	–

^*^P < 0.05

To further explore the meaningful statistical results, we used 12 weeks as the node to divide the training period into three subgroups including less than 12 weeks, 12 weeks and more than 12 weeks to perform the network meta-analysis again. The probabilistic ranking is shown in Table 3.

**Table 3:** Probability ranking

Outcome indicators	Training period	Rank 1	Rank 2	Rank 3	Rank 4
FPG	< 12 w	N	Baduanjin	Taijiquan	
	12 w	N	Taijiquan	Baduanjin	Yijinjing
	> 12 w	N	Baduanjin	Taijiquan	
HbAlc	< 12 w	N	Wuqinxi	Baduanjin	Taijiquan
	12 w	N	Baduanjin	Taijiquan	Yijinjing
	> 12 w	N	Baduanjin	Taijiquan	
2hPG	< 12 w	N	Taijiquan	Baduanjin	
	12 w	N	Baduanjin	Taijiquan	
	> 12 w	N	Baduanjin	Taijiquan	
TC	< 12 w	N	Wuqinxi	Baduanjin	
	12 w	Baduanjin	N	Taijiquan	Yijinjing
	> 12 w	N	Taijiquan	Baduanjin	
TG	< 12 w	N	Wuqinxi	Baduanjin	
	12 w	N	Baduanjin	Yijinjing	Taijiquan
	> 12 w	N	Taijiquan	Baduanjin	
HDL	< 12 w	Baduanjin	Wuqinxi	N	
	12 w	Yijinjing	Taijiquan	Baduanjin	N
	> 12 w	Baduanjin	Taijiquan	N	
LDL	< 12 w	N	Wuqinxi	Baduanjin	
	12 w	N	Baduanjin	Yijinjing	Taijiquan
	> 12 w	N	Baduanjin	Taijiquan	

*w* weeks, *N* no traditional exercise intervention

Thirty studies reported HbA1c changes involving four traditional exercise therapies, resulting in a total of ten pairwise comparisons. The MD in the change of HbAlc levels was 0.37 point lower in the Taijiquan group than in the no traditional exercise intervention group (MD = 0.37; 95% CI 0.24 to 0.58; 926 participants; *P* < 0.05) and 0.45 point lower in the Baduanjin group (MD = 0.45; 95% CI 0.32 to 0.62; 1325 participants; *P* < 0.05). The difference between the other pairwise comparisons was not statistically significant (*P* > 0.05; Table 2). Probability ranking resulted in the conclusion that Taijiquan has the best effect on reducing HbAlc levels (Fig. 5). The results of subgroup analysis showed that Baduanjin had better effects than Wuqinxi (MD =  − 0.60, 95% CI − 1.71 to 0.52, *P* < 0.05) and Taijiquan had better effects than Wuqinxi (MD =  − 0.73, 95% CI − 2.03 to 0.66, *P* < 0.05) when the training period was less than 12 weeks. Baduanjin had better effects than Taijiquan (MD = 0.02, 95% CI − 0.84 to 0.86, *P* < 0.05) when the training period was 12 weeks. The specific probability rankings are shown in Table 3.

Thirteen studies reported 2hPG changes involving two traditional exercise therapies, resulting in a total of three pairwise comparisons. The MD in the change of 2hPG levels was 0.15 point lower in the Taijiquan group than in the no traditional exercise intervention group (MD = 0.15; 95% CI 0.06 to 0.42; 479 participants; *P* < 0.05) and 0.36 point lower in the Baduanjin group (MD = 0.36; 95% CI 0.16 to 0.76; 663 participants; *P* < 0.05). Although the difference between the other pairwise comparisons was not statistically significant (*P* > 0.05; Table 2), we can infer from the existing evidence that Taijiquan has the best effect on reducing 2hPG levels. Taking 12 weeks as the node, the studies containing 2hPG data were divided into three subgroups according to the training time: less than 12 weeks, 12 weeks and more than 12 weeks, and network meta-analysis was performed again. None of the comparisons was statistically significant. The probability ranking is shown in Table 3.

### Secondary outcomes

Nineteen studies reported TC changes involving four traditional exercise therapies, resulting in a total of ten pairwise comparisons. The MD in the change in TC was 0.57 point lower in the Baduanjin group than in the no traditional exercise intervention group (MD = 0.57; 95% CI 0.45 to 0.72; 922 participants; *P* < 0.05). The probabilistic ranking showed that Yijinjing has the best effect on reducing TC levels (Fig. 5). The results of subgroup analysis showed that Baduanjin had better effects than Wuqinxi when the training period was less than 12 weeks (MD =  − 0.77, 95% CI − 2.08 to 0.60, *P* < 0.05) and 12 weeks (MD =  − 0.16, 95% CI − 0.81 to 0.44, *P* < 0.05). The specific probability rankings are shown in Table 3.

Nineteen studies reported TG changes involving four traditional exercise therapies, resulting in a total of ten pairwise comparisons. The MD in the change in TG was 0.46 point lower in the Baduanjin group than in the no traditional exercise intervention group (MD = 0.46; 95% CI 0.3 to 0.72; 753 participants; *P* < 0.05). The probabilistic ranking concludes that Baduanjin has the best effect on reducing TG levels (Fig. 5). The results of subgroup analysis showed that Baduanjin had better effects than Wuqinxi when the training period was less than 12 weeks (MD =  − 0.18, 95% CI − 0.75 to 0.38, *P* < 0.05) and 12 weeks (MD = 0.99, 95% CI − 2.22 to 0.26, *P* < 0.05). Taijiquan had better effects than Yijinjing (MD =  − 0.23, 95% CI − 1.47 to 0.71, *P* < 0.05) when the training period was 12 weeks. The specific probability rankings are shown in Table 3.

Fifteen studies reported LDL-C changes involving four traditional exercise therapies, resulting in a total of ten pairwise comparisons. The probabilistic ranking showed that Taijiquan has the best effect on reducing LDL-C levels (Fig. 5). The results of subgroup analysis showed that Baduanjin had better effects than Wuqinxi (MD =  − 0.26, 95% CI − 0.85 to 0.34, *P* < 0.05) when the training period was less than 12 weeks. Taijiquan had better effects than Yijinjing (MD =  − 0.22, 95% CI − 1.25 to 0.85, *P* < 0.05) when the training period was 12 weeks. The specific probability rankings are shown in Table 3.

Twenty-one studies reported HDL-C changes involving four traditional exercise therapies, resulting in a total of ten pairwise comparisons. The MD in the change in HDL-C was 1.18 point lower in the Baduanjin group than in the no traditional exercise intervention group (MD = 1.18; 95% CI 1.08 to 1.29; 922 participants; *P* < 0.05). It is inferred from the change in the MD value that Baduanjin has the most obvious effect in improving HDL-C levels. The results of subgroup analysis showed that Baduanjin had better effects than Wuqinxi (MD =  − 0.26, 95% CI − 0.06 to 0.57, *P* < 0.05) when the training period was less than 12 weeks. Baduanjin had better effects than Taijiquan (MD =  − 0.03, 95% CI − 0.14 to 0.12, *P* < 0.05) and Yijinjing (MD =  − 0.07, 95% CI − 0.25 to 0.15, *P* < 0.05) when the training period was 12 weeks. Taijiquan had better effects than Yijinjing (MD =  − 0.04, 95% CI − 0.22 to 0.15, *P* < 0.05) when the training period was 12 weeks. Baduanjin had better effects than Taijiquan (MD =  − 0.04, 95% CI − 0.22 to 0.15, *P* < 0.05) when the training period was more than 12 weeks. The specific probability rankings are shown in Table 3.

Two studies reported ISI changes involving two exercise therapies, resulting in a total of three pairwise comparisons. There was no statistically significant difference between the pairwise comparisons (*P* > 0.05) (Table 2). The probability ranking was as follows: Taijiquan > Baduanjin > no traditional exercise intervention (Fig. 5).

### Publication bias analysis

**Fig. 6:**
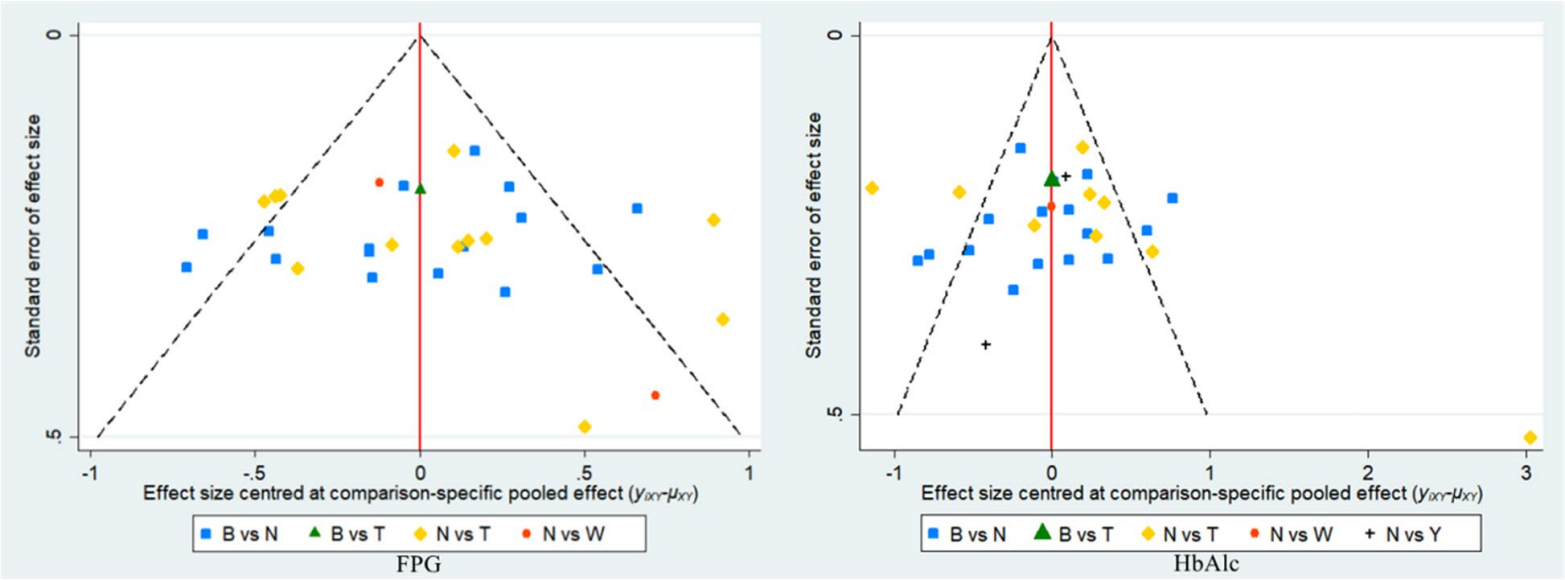
Correction-comparison funnel chart (FPG and HbAlc)

Taking FPG and HbAlc as a representative, as shown in Fig. 6, the correction-comparison funnel chart is basically symmetrical, and the possibility of publication bias is low.

## Discussion

### Principal findings

Traditional Chinese exercises are aerobic exercises with a strong theoretical basis in Chinese medicine and a long history. Such exercises are nondrug therapies guided by the theories of Chinese medicine [29]. At present, an increasing number of studies have confirmed that long-term regular and moderate traditional Chinese exercise has obvious effects on controlling diabetes and lowering blood sugar and can significantly reduce HbAlc, FPG and 2hPG levels and other disease indicators to improve the quality of life of patients.

The network meta-analysis results show that Taijiquan had the best overall effect, especially in improving HbAlc, 2hPG and LDL-C levels and ISI, compared with the other three traditional exercise therapies. Yijinjing was more effective in improving FPG and TC levels. Baduanjin showed better results in improving TG and HDL-C levels. After subgroup analysis, it was found that when the training period was less than 12 weeks, Taijiquan had the best probability of improving FPG and HbA1c levels, and Baduanjin had the best ranking for improving 2hPG, TC, TG, HDL-C and LDL-C levels; when the training period was 12 weeks long, Taijiquan and Yijinjing were effective for improving blood glucose indices and blood lipid indices. Taijiquan had the best probability of improving TG, 2hPG and LDL-C levels, and Yijinjing had the best ranking for improving FPG, HbAlc, TC and HDL-C levels; when the training period was more than 12 weeks, Taijiquan was the best in improving blood glucose indicators and LDL-C levels. The probability ranking of Baduanjin was the best in improving blood lipid indices such as TC, TG and HDL-C levels.

Taijiquan is a complex motor skill consisting of a series of fine and coherent movements with hand and foot coordination, characterized by slow rhythm, moderate intensity and unity of body and mind, which has a significant effect on improving HbAlc, 2hPG, LDL-C and ISI indicator levels, ranking first among the four traditional exercise therapies [30]. Taijiquan exercise can effectively promote the body’s consumption of blood sugar; effectively improve the intake, oxidation and utilization of sugar by the patient’s muscles; and ultimately achieve the goal of blood sugar control [31]. The Asia–Pacific Diabetes Prevention and Control Guidelines clearly stipulate that HbAlc is the internationally recognized “gold standard” for diabetes monitoring [32]. The decrease in the HbA1c index reflects that Taijiquan exercise can effectively increase the stability of the disease and delay the occurrence and development of atherosclerosis and other similar complications. LDL-C is the primary atherogenic lipoprotein among all serum lipoproteins, and as an important detection index in blood lipids, their reduction showed that Taijiquan exercise therapy can improve lipid metabolism, accelerate the decomposition of fat and the utilization of fat cholesterol and free fatty acids, reduce the body’s lipid peroxidation reaction, reduce blood lipid and blood sugar interactions with each other and reduce cardiovascular and cerebrovascular risk factors. Taijiquan exercises also have the best effects in the existing studies in improving the ISI, improving the body’s sensitivity to insulin, enhancing the binding of insulin and cell receptors and reducing insulin resistance.

Yijinjing, as a traditional Chinese sport that combines physical activity, breathing, breathing regulation and psychological regulation, has the effect of strengthening the body and preventing diseases [33]. Among the four traditional Chinese exercise therapies, Yijinjing has the best effect on reducing blood glucose and lipid indexes such as FPG, TC, TG and LDL-C in T2DM patients. Yijinjing can promote glandular secretion, can regulate the body’s blood sugar and lipid metabolism, can effectively prevent the occurrence of cardiovascular diseases and is simple and easy to learn [34, 35]. However, since only two of the included studies focused on the efficacy of Yijinjing, this conclusion should be carefully considered. More research is needed in the future to prove it. FPG is an important indicator reflecting the function of pancreatic β-cells. The decrease in FPG levels indicates that Yijinjing can improve the function of pancreatic β-cells, promote the secretion of insulin and regulate blood glucose metabolism. Disorders of blood glucose metabolism may increase blood lipids and further affect islet function. TC and TG levels are common causes of cardiovascular and cerebrovascular complications in type 2 diabetes. Baduanjin exercise therapy had the best probability ranking in increasing the levels of TG and HDL-C. HDL-C has the effect of preventing atherosclerosis and reducing the mortality rate of coronary heart disease.

### Limitations of the study

The inclusion of only RCTs in this study and the absence of its own precontrol and postcontrol studies may have affected the accuracy and comprehensiveness of the results to some extent. The methodological quality of the literature included in this study is generally low. Only 15 of the 33 studies used random number tables for random allocation. Only five articles mentioned blinding and all using single-blind methods, and most of the studies on allocation plan hiding and blinding were not mentioned. Although the number of people who dropped out or were lost to follow-up was not included in the analysis results, there may still be biases that affect the authenticity of the results. It is recommended to conduct a more rigorous and scientific, multicentre, large-sample, randomized controlled study. The Wuqinxi and Yijinjing movement therapies involved less research, and the extrapolation of their results may be poor. It is suggested that more research on the Wuqinxi and Yijinjing movement therapies should be carried out in the future. The results of this study were mainly derived from Chinese patients, and more studies are needed to validate the results in groups other than Chinese patients.

## Conclusion

This study used the method of network meta-analysis to analyse four traditional Chinese exercise therapies. In general, Taijiquan exercises have significant advantages. Taijiquan focuses on reducing blood lipids and blood sugar, which can reduce cardiovascular and cerebrovascular risk factors and slow down the progression of the disease. After subgroup analysis, it was found that when the training period was less than 12 weeks, Baduanjin had better effects in improving 2hPG, TC, TG, HDL-C and LDL-C indicator levels. Taijiquan was more effective than Yijinjing in reducing FPG levels. When the training period was 12 weeks, the effect of Yijinjing in improving FPG, HbAlc, TC and HDL-C levels was better than other traditional Chinese exercise, and Taijiquan had better effects in improving 2hPG, TG and LDL-C indicator levels. When the training period was longer than 12 weeks, Taijiquan had better effects in improving FPG, HbAlc, 2hPG and LDL-C indicator levels, and Baduanjin had better effects in improving TC, TG and HDL-C indicator levels. Therefore, in clinical practice, suitable traditional exercises can be selected for exercise and treatment according to the different types of patients, disease bias and training cycles.

## Data Availability

Data are available in a public, open access repository.

## References

[1] American Diabetes Association (2019). 2. Classification and diagnosis of diabetes: standards of medical care in diabetes-2019. Diabetes Care.

[2] Chinese Diabetes Society. Guidelines for the prevention and control of type 2 diabetes in China (2017 Edition). Chin J Pract Intern Med 2018;38:292-344.

[3] Xinyue L, Qian L, Guiying S, Yiying H, Xuepei L, Lin B (2020). Research progress on stem cell therapy for type 2 diabetes mellitus and associated complications. Chin J Comparative Med.

[4] Qin Z. Nursing common sense to prevent complications of type 2 diabetes. Special health 2020;106.

[5] Xingchen Y, Yaling Z, Jingge G (2021). Clinicopathological characteristics and prognosis of minimal change nephropathy with type 2 diabetes mellitus. Chin Gen Pract.

[6] Sun H, Saeedi P, Karuranga S, Pinkepank M (2022). IDF diabetes atlas: global, regional and country-level diabetes prevalence estimates for 2021 and projections for 2045. Diabetes Res Clin Pract.

[7] Guideline for the prevention and treatment of type 2 diabetes mellitus in China (2020 edition) (Part 1). Chin J Pract Intern Med 2021;41;08:668–695.

[8] Balducci S, Sacchetti M, Haxhi J (2014). Physical exercise as therapy for type 2 diabetes mellitus. Diabetes Metab Res Rev.

[9] Guanzhou H, Chenji W, Jiang H (2009). The effect of long-term aerobic exercise on the metabolism of blood glucose and lipids in patients with type 2 diabete. Chin J Physical Med Rehab.

[10] Jiangtao F, Dong W (2022). On the concept of Taijiquan. J Handan Univ.

[11] Huihui W, Zhongqiu J, Zihua Z (2019). Biomechanical characteristics of lower limbs in 24-form Taichi movement. J Chengdu Sport Univ.

[12] Yunfeng C, Surong L, Rui L (2015). Effect of Health Qigong Baduanjin on the pulmonary function of patients with chronic obstructive pulmonary disease in stable period. Chin Med Modern Distance Educ China.

[13] Shengwei D, Zhidong C, Tonggang F (2021). Historical development and meaning of “Wuqinxi” health preservation culture. J Wuhan Sports Univ.

[14] Qiaoju Y, Linqi Y (2018). Research progress on the effect of Yijinjing on limb function in patients with rheumatoid arthritis. Chin Med Modern Distance Educ China.

[15] Jiaqi Z, Yunchuan W (2012). Exercise therapy and type 2 diabete. World J Integrated Traditional and Western Med.

[16] Yongjia S, Jiyuan Z, Zejia L (2015). Traditional theory of exercise therapy in the modern value of chronic diseases prevention and control. Clin J Tradit Chin Med.

[17] Fan W, Enfeng S, Yan B, et al. Taijiquan for the treatment of type 2 diabetes. Clin J Tradit Chin Med 2010;205–7.

[18] Xilin L, Yingjie W, Dan R (2019). Analysis of the effect of “5123 Baduanjin” on blood glucose metabolism homeostasis in high-risk populations of type 2 diabetes. Asia-Pacific Traditional Med.

[19] Xinghai L. Research on Health Qigong•Wuqinxi on hemorheology of type 2 diabetic patients. Journal of Liaoning Normal University (Nature Science Edition) 2007;369–71.

[20] Xuejuan M, Linjun Z, Xuezhen F (2019). Analysis of the clinical effect of traditional fitness exercises•Yijinjing on type 2 diabetes. J Front Med.

[21] Chinese Diabetes Society. Guidelines for the prevention and control of type 2 diabetes in China (2010 Edition). Chin J Diabetes 2012;20:81–117.

[22] Chinese Diabetes Society. Guidelines for the prevention and control of type 2 diabetes in China (2013 Edition). Chin J Diabetes 2014;22:2–42.

[23] Zaiying L, Nanshan Z (2008). Internal medicine.

[24] Hongqiu G, Yang W, Wei L (2014). Application of Cochrane bias risk assessment tool in meta-analysis of randomized controlled study. Chin Circulation J.

[25] Jie M, Ying L, Laiping Z (2012). Application and comparison of Jadad scale and Cochrane bias risk assessment tool in quality evaluation of randomized controlled trials. China J Oral and Maxillofac Surg.

[26] Chao Z, Feng S, Xiantao Z (2014). R software calls JAGS software to realize network meta-analysis. Chin J Evid Based Med.

[27] Dan W, Junxia ZH, Zhenyun M (2009). Heterogeneity and its treatment in meta analysis. Chin J Evid Based Med.

[28] Gert van Valkenhoef, Tommi Tervonen, Tijs Zwinkels, et al. ADDIS: a decision support system for evidence-based medicine. 2013, 55;2:459–75.

[29] Dongmei Y, Xiangdi L, Zhihong L (2017). Research progress of traditional Chinese medicine exercise therapy on diabetes mellitus. Clin J Chin Med.

[30] Lin L, Xiaoyou Z, Yakui Xu, et al. Dynamic changes in brain function during the early stages of Taijiquan skill learning: an fMRI study based on motor representations. Acta Psychologica Sinica 2023;55;8;1243–1254.

[31] Xiaobing L (2013). The effect of Taijiquan exercise on the oxidative stress and inflammation levels in elderly patients with type 2 diabetes. Chin J Gerontol.

[32] Xiaojie F, Lin W (2016). Clinical value of serum C peptide and glycosylated hemoglobin test in diagnosis of diabetes mellitus. Diabetes New World.

[33] Xiaoyan L, Weimin H, Yonghong W (2022). Observation of therapeutic effect of Yijinjing exercise on elderly patients with type 2 diabetes. Shanghai Med Pharm J.

[34] Yongyi L, Hong T, Youhua W (2014). Combination of changing tendon exercise and dietary therapy for impaired glucose regulation. Shanghai J Tradit Chin Med.

[35] Yufeng S, Xiaodan L (2012). Effect of fitness QigongYi Jinjing on physical function and blood lipid of the elder people. J Nanjing Sports Institute.

